# Personalized [177Lu]Lutetium-PSMA Therapy for Patients with Pre-Treated Castration-Resistant Prostate Cancer: A Single Institution Experience from a Comprehensive Cancer Centre

**DOI:** 10.3390/cancers15123216

**Published:** 2023-06-16

**Authors:** Wolfgang Thaiss, Friedemann Zengerling, Julia Friedrich, Veronika Hechler, Michael Grunert, Christian Bolenz, Thomas Wiegel, Ambros J. Beer, Vikas Prasad

**Affiliations:** 1 Department of Nuclear Medicine, University Hospital Ulm, 89081 Ulm, Germany; 2 Department of Diagnostic and Interventional Radiology, University Hospital Ulm, 89081 Ulm, Germany; 3 Surgical Oncology Ulm, i2SOUL Consortium, 89075 Ulm, Germany; 4 Department of Urology and Pediatric Urology, University Hospital Ulm, 89081 Ulm, Germany; 5 Department of Nuclear Medicine, German Armed Forces Hospital of Ulm, 89081 Ulm, Germany; 6 Department of Radiation Oncology, University Hospital Ulm, 89081 Ulm, Germany; 7 Division of Nuclear Medicine, Mallinckrodt Institute of Radiology, Washington University in Saint Louis, Saint Louis, MO 63130, USA

**Keywords:** castration-resistant prostate cancer, lutetium PSMA, radioligand therapy, prediction, PSA response

## Abstract

**Simple Summary:**

Advanced prostate cancer can be treated with [177Lu]-Lutetium-PSMA radi-oligand therapy (Lu-PSMA) in advanced stages. However, little is known about the predictive factors for treatment success. We retrospectively analyzed Lu-PSMA radioligand treatments in 86 patients. The focus of the study was to describe clinical factors at baseline and during early treatment that are related to survival. In addition, imaging results from PSMA PET/CT-, laboratory values such as PSA-response, and safety and tolerability of the treatment were assessed. The observed side effects were comparable to previous studies and anemia was the most frequent adverse event. Pre-treatment hemoglobin level of ≥10 g/dL and a lower PSA values at treatment start were favorable factors for longer survival. The presence of visceral or liver metastases were not significantly associated with worse prognoses. With careful patient selection, an individualized Lu-PSMA treatment approach is feasible and patients with dose-limiting factors or visceral metastases should be included in prospective trials. This novel information can be helpful for therapeutic decision making.

**Abstract:**

Castration resistant prostate cancer (CRPC) is characterized by an aggressive biological behavior with a relatively short survival time, especially in progressive tumors pretreated with new hormonal agents and taxane chemotherapy. [177Lu]-Lutetium-PSMA (Lu-PSMA) treatment has proven efficacy in these patients. However, around 30% of the CRPC patients do not benefit from Lu-PSMA treatment, and little is known about predictive factors for treatment success if Lu-PSMA is offered in an individualized approach based on clinical and laboratory features. In this monocentric retrospective study, 86 CRPC patients receiving Lu-PSMA treatment were evaluated. The focus of the study was to describe clinical factors at baseline and during early treatment that are related to overall survival (OS). In addition, PSMA PET/CT-, PSA-response, and safety and tolerability (CTCAE adverse event reporting) were assessed. Efficacy endpoints were calculated using stratified Kaplan–Meier methods and Cox regression models. Mean applied dose was 17.7 GBq (mean 5.3 ± 1.1 GBq per cycle) with an average of 3.6 (range 1–8) therapy cycles. Patients were followed up for a mean of 12.4 months (range 1–39). The median OS was 15 months (95% CI 12.8–17.2). The best overall response rate in patients assessed with PSMA PET/CT and PSA response was 27.9%, and 50.0% had at least stable disease. Nine patients had a ≥grade 3 adverse event with anemia being the most frequent adverse event. Positive predictors for prolonged OS from baseline parameters were pre-treatment hemoglobin level of ≥10 g/dL and a lower PSA values at treatment start, while the presence of visceral or liver metastases were not significantly associated with worse prognoses in this cohort. With careful patient selection, an individualized Lu-PSMA treatment approach is feasible and patients with dose-limiting factors or visceral metastases should be included in prospective trials.

## 1. Introduction

Metastatic castration resistant prostate cancer (mCRPC) is characterized by an aggressive biological behavior and a relatively short survival time, especially in cases of progressive disease after treatment with both new hormonal agents and taxane chemotherapy [1]. The introduction of [177Lu]Lutetium-PSMA (Lu-PSMA) treatment into clinical practice and guidelines [2] has gained momentum over recent years [3,4,5], and today it forms a viable treatment option for mCRPC patients, offering as much as a ~50% response rate in a recent phase III trial [6]. However, around 30% of mCRPC patients do not benefit from Lu-PSMA treatment, and little is known about predictive factors for the success of the treatment. Additionally, the optimal line of therapy for Lu-PSMA during disease progression is still under investigation, with recent studies suggesting beneficial outcomes when it is administered earlier during the course of the disease, and the results have also been promising in comparison to chemotherapeutic regimens when careful patient selection was applied [7]. Moreover, the selection criteria for eligible patients have also been under continuous investigation as the larger trials excluded certain patients—e.g., those with elevated liver enzymes due to hepatic metastases or impaired renal function. Based on the results of large phase II and phase III studies, Lu-PSMA therapy has been approved by official agencies in several countries, broadening the clinical introduction and addressing reimbursement aspects [6,8]. Several ongoing studies will supplement these results with the assessment of first-line and combination therapies [9,10,11]. Meanwhile, the availability of Lu-PSMA treatment in selected countries and its introduction into the current guidelines has led to the availability of real-world patient data outside clinical studies.

In this study, we retrospectively evaluated charts of patients treated with at least one therapy cycle of Lu-PSMA (PSMA I&T or PSMA-617). The dose and the dosing interval varied to some extent based on the clinical and the laboratory parameters of the patients. The decision for therapy was made by the local tumor board. The survival data of patients, therapy limiting factors, and side effects in an individualized therapeutic concept were all observed in this study. 

## 2. Materials and Methods

A retrospective analysis was performed within a single center cohort consisting of adult patients with histologically confirmed, progressive metastatic castration-resistant prostate cancer treated with Lu-PSMA, treated in Ulm University Hospital October 2017 and August 2021. 

For radiolabeling, PSMA-617 was obtained from ABX Advanced Biochemical Compounds (Radeberg, Germany), PSMA I&T was obtained from Scintomics, and Lu-177 was obtained from ITM (Munich, Germany). Radiolabeling was performed in our institution’s local GMP radiopharmacy according to the methods previously described. RLT with 177Lu-PSMA was performed under the national regulations of compassionate use of a non-approved drug according to AMG §13.2b (German Medicinal Product §13.2b), and Lu-PSMA was administered with a standard dose of 6 GBq per cycle [12]. Patients with impaired renal, hepatic, or bone marrow function received Lu-PSMA in modified doses lower than 6 GBq, as per the local standard and also after the agreement of two nuclear medicine physicians with more than 15 years of experience in radioligand therapy (V.P., A.J.B.). Prior to the initiation of Lu-PSMA treatment, the indication for Lu-PSMA therapy was approved by the institutional multidisciplinary tumor board panel. If Lu-PSMA was administered as part of a combination therapy other than androgen deprivation therapy, patients were excluded from our analysis.

The decision to undertake Lu-PSMA therapy was made in the local Comprehensive Cancer Center tumor board for prostate cancer, and it was based on the following factors:

- Documented progressive mCRPC after at least one line of systemic therapy for mCRPC after ADT.
- Evidence of PSMA positive metastases on PSMA PET/CT without any PSMA negative suspicious lesions on CT.
- In case of discrepancy between PSMA, PET, and CT, the lesions, if amenable for biopsy, were confirmed either by histopathology or with best supporting evidence.
- An Eastern Cooperative Oncology Group (ECOG) performance status up to 3.
- Hb > 6g/dL, Thrombocytes > 50 × 10^9^/L. In patients with values lower than the aforementioned cut-offs, either no Lu-PSMA therapy was offered or, depending on the ECOG status of the patients, they went for pre Lu-PSMA therapy blood transfusion (erythrocytes or thrombocytes without any G-CSF or erythropoietin)
- In patients with brain metastases and/or intraspinal metastases and/or painful bone lesions and/or the presence of acute pathological fracture or a suspicion of a high probability of bone-related events from the lesions, Lu-PSMA therapy was preceded by external beam radiation therapy (EBRT). After EBRT, patients were treated with Lu-PSMA within 3–4 weeks. In patients with mild pain without any need urgent need for opiates or pain palliation with EBRT, Lu-PSMA therapy was started first, and it was then followed by EBRT within 1–2 weeks.
- In patients with evidence of bone marrow or renal function deterioration or diffuse liver metastases with elevated GGT and/or Bilirubin, the dose of Lu-PSMA therapy was reduced by 25–75%.
- In patients with high tumor burden and/or evidence of rapid tumor kinetic on pre-therapy PSMA, PET/CT, or PSA value or clinical symptoms, the time interval between two treatment cycles was reduced from 6 weeks to 4–5 weeks.
- Patients were intended to be treated with a minimum of 4 Lu-PSMA cycles. However, the decision to carry on the 3rd and the higher therapy cycles was assessed in MDT after every 2 therapy cycles. Apart from PSA response, improvement/deterioration in clinical symptoms, clinical need, ECOG status, posttherapy scans including SPECT/CT, and PSMA PET/CT response were also all considered when making the decision of whether to continue or discontinue treatment.

All patients were treated and monitored according to local clinical practice. All patients that did not continue treatment at any point were included in the analysis. The primary outcome of interest was overall survival (OS). In addition, PSA response (confirmed PSA decrease ≥ 50%) and best overall PSA response were assessed. Additionally, PSMA-PET/CT response was analyzed, as previously described [13]. Safety and tolerability represented by the rate of CTCAE grade ≥3 events were also documented for the patients’ charts. Exploratory analysis included clinical and biochemical predictors for improved OS.

The retrospective chart review was conducted according to the Good Clinical Practice guidelines of the International Conference on Harmonization and the principles of the Declaration of Helsinki. The study was approved by the Ethics Committee of Ulm Univer-sity Ulm University on 12 October 2020 and 22 October 2021 (reference 195/20).

Statistical analyses were performed with SPSS (IBM SPSS Statistics for Windows, Version v.28.0.1. Armonk, NY, USA: IBM Corp.). Summary descriptive statistics were applied to baseline patient characteristics. OS were estimated by the Kaplan–Meier method and tested with log-rank tests. Multivariate Cox regression analysis was performed for baseline characteristics (VISION criteria met, presence of visceral metastases, liver metastases, haemoglobin, and PSA values at therapy start) to assess the association of patient characteristics with respect to OS. All tests were two-sided, and *p*-values < 0.05 were considered significant.

## 3. Results

### 3.1. Characteristics of the Study Cohort

A total of 86 consecutive patients with mCRPC who underwent Lu-PSMA treatment between October 2017 and August 2021, meeting the criteria for treatment as mentioned above, had a minimum of 6 months follow-up after last cycle of therapy (unless patient deceased prior to it), were evaluated in this retrospective cohort study. All of the included patients were pretreated with androgen deprivation therapy and at least one line of systemic therapy for mCRPC. A total of 81 patients (94.2%) were pretreated with at least one line of new hormonal agent (e.g., abiraterone or enzalutamide) and 65 patients (75.6%) had prior taxane treatment (docetaxel or cabazitaxel). The median CRPC therapy line in which Lu-PSMA was applied was third line. Further clinically relevant baseline characteristics are depicted in Table 1 (age, ECOG, site of disease (bone, lymph node, and visceral metastases)), PSA at treatment initiation, glomerular filtration rate (GFR) (mL/min), serum alkaline phosphatase (ALP) and serum lactate dehydrogenase (LDH) at treatment initiation, Gleason Score at diagnosis, previous local therapy to the primary, previous new hormonal agent, and previous taxane therapy.

### 3.2. Treatment Exposure and Tolerability

After a mean follow-up time of 12.4 months (range 1–39), the mean number of applied Lu-PSMA cycles (administration every 6 ± 2 weeks) was 3.6 (range: 1–8). The mean applied activity of Lu-PSMA per cycle was of 5.3 ± 1.1 GBq (range 2–6 GBq). Dose reductions (doses were reduced by 25% or 50%) due to adverse events were necessary in 21 (24.4%) of the patients treated with Lu-PSMA. Grade 3–4 adverse events according to the Common Terminology Criteria for Adverse Events (CTCAE) classification occurred in nine (10.3%) of the patients with anemia, and were the most common cases in AE. Further adverse events are depicted in Table 2. Based on the investigator’s assessment, the most common reasons for treatment discontinuation were disease progression (*n* = 24), death (*n* = 49: of those, 12 within 90 days after treatment, and 3 with potential treatment-related adverse events), completion of the planned number of cycles (*n* = 10), or the patient’s wish (*n* = 3).

### 3.3. PSMA-PET/CT Response

**Figure 1:**
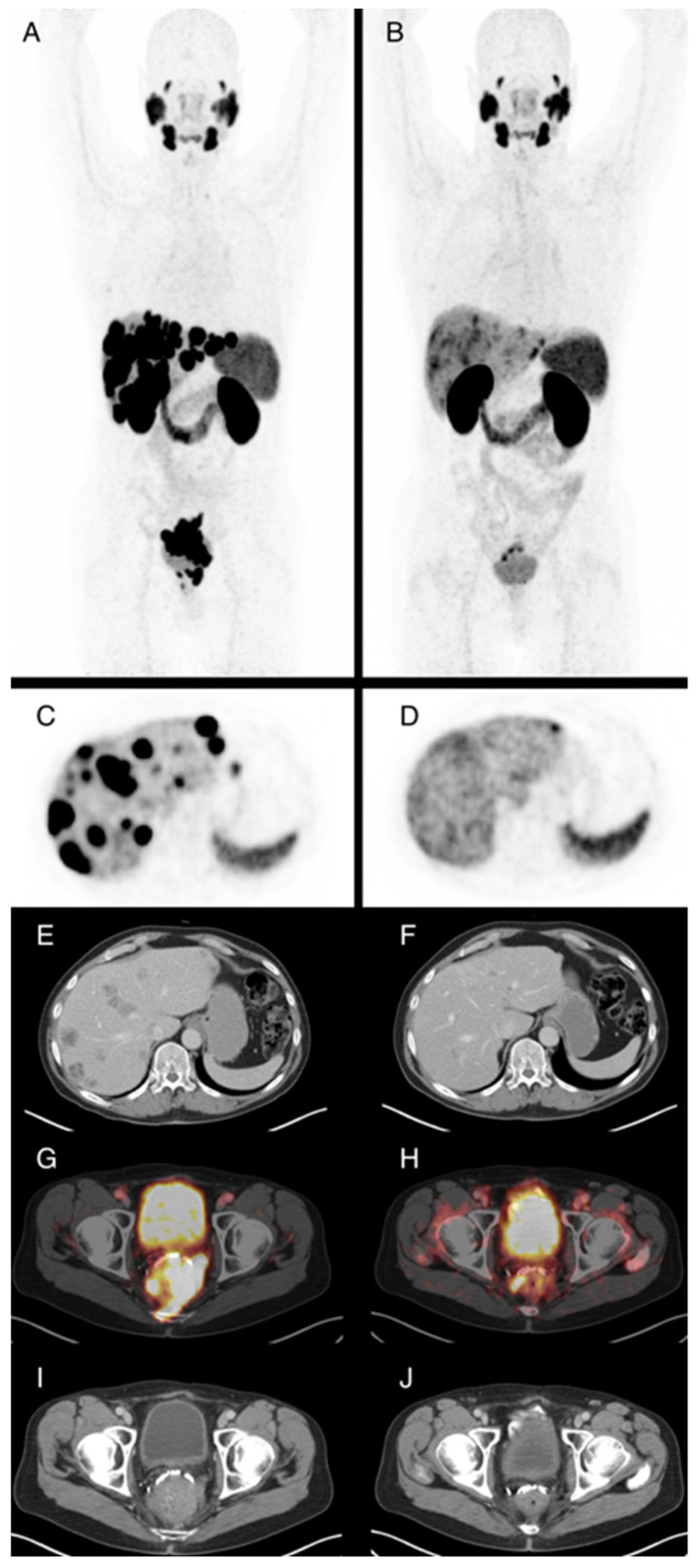
61-year-old patient with locally advanced prostate cancer and metastases in lymph nodes, bone, and liver. Maximum intensity projections of Ga-68-PSMA-PET/CT before (**A**) and after ((**B**), SUVmax. 15) four cycles of Lu-177-PSMA radioligand therapy (RLT) with application of 23.6 GBq in total, which demonstrate very good partial response. PSA levels were 25.5 ng/mL prior to RLT and 0.04 ng/mL after the fourth cycle. Axial PET images of the liver before (**C**) and after (**D**) therapy show reduction of PSMA-expression of the liver metastases, associated with subtotal reduction of hypodense liver metastases in contrast enhanced CT (**E**,**F**). The locally advanced primary cancer also shows substantially reduced PSMA expression of the tumor, with infiltration of the rectum in fusion images (**G**,**H**) and contrast enhanced CT images (**I**,**J**) from baseline to staging after four cycles of RLT.

**Figure 2:**
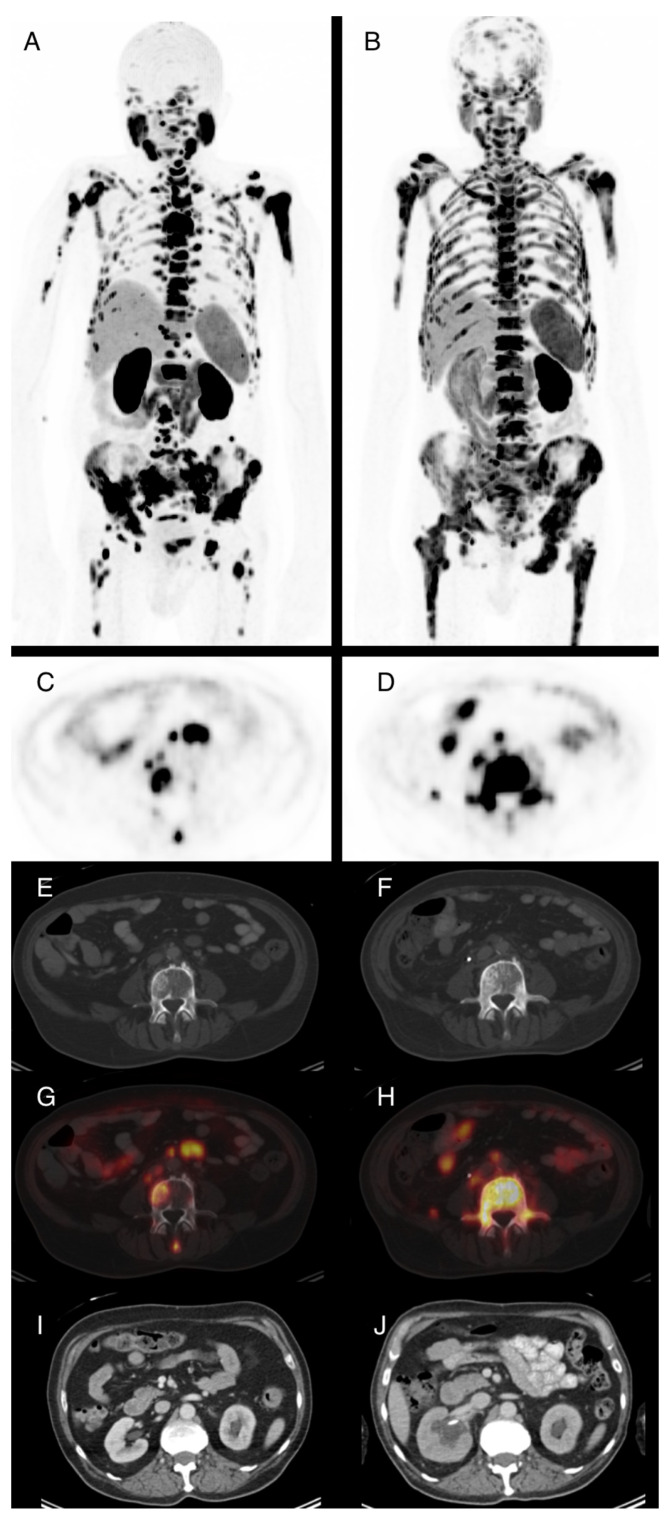
74-year-old patient with locally recurrent prostate cancer and metastases in lymph nodes and bone. Maximum intensity projections of F-18-PSMA-PET/CT before (**A**) and after ((**B**), SUVmax. 15) two cycles of Lu-177-PSMA radioligand therapy (RLT) with application of 10.7 GBq total demonstrate progressive disease. PSA levels were 169 ng/mL prior to RLT and 233 ng/mL after two cycles. Axial PET images show increasing PSMA-expression in the L4 vertebra (**C**,**D**) that is not totally reflected in corresponding CT images with sclerotic bone lesions (**E**,**F**). (**G**,**H**) show image fusion of PET and CT at the same position as (**E**,**F**). Due to progressive metastatic obstruction of the right ureter, postrenal kidney failure with hydronephroses occurred and a ureteral stent was placed (**J**, compared to **I**). This also explains the reduced tracer excretion via the right kidney observed in (**B**).

Based on PSMA PET/CT, 27 of 86 patients had partial response following Lu-PSMA treatment, corresponding to a best overall response rate of 31.4%. No complete response was observed in our series. Eleven patients had at least stable disease as the best overall response. In 24 patients, no PSMA PET/CT could be obtained after at least two cycles of therapy because these patients died before imaging. A total of 24 patients showed progressive disease in PSMA PET/CT after two cycles of therapy. Patient examples are given in Figure 1 and Figure 2.

### 3.4. PSA Response

In our study cohort, 73 of 86 (85%) patients had pre- and post-therapeutic PSA assessments available to them. A total of 29 of these patients had a PSA decline of at least 50% under therapy, and 38 had rising PSA values during Lu-PSMA treatment with a median time to PSA progression of 57 days (range 28–938).

### 3.5. Survival Analysis

**Figure 3:**
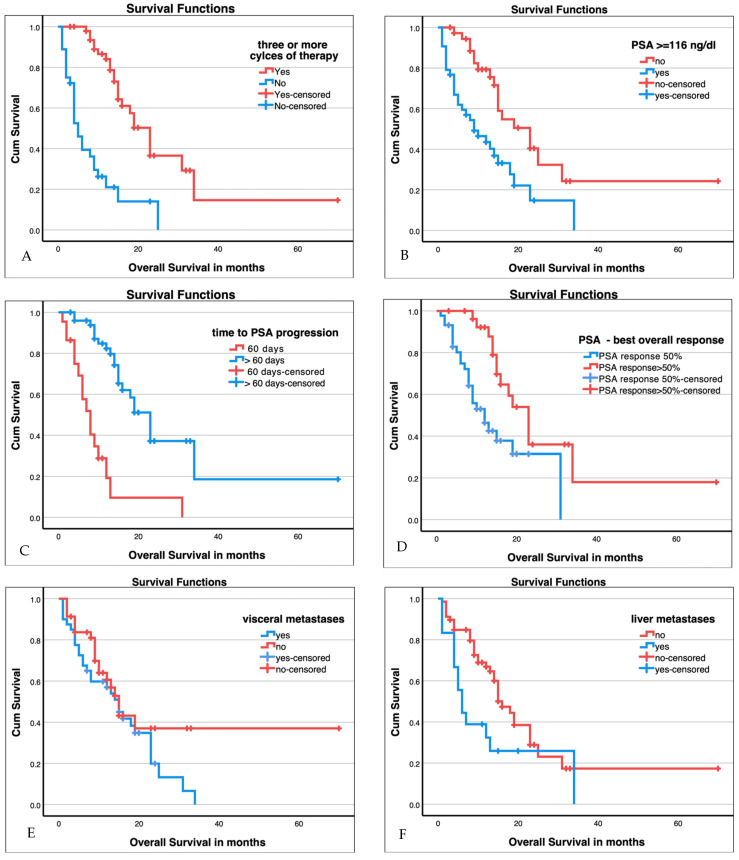

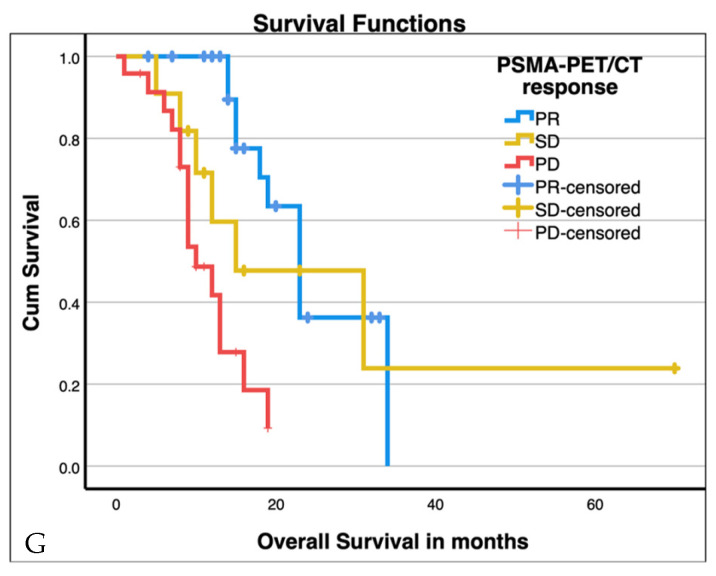
Kaplan–Maier plots for patients with three or more cycles of therapy ((**A**), *p* < 0.001), baseline PSA ≥ 116 ng/mL (median, (**B**), *p* > 0.001), time to PSA progression ((**C**), *p* < 0.001), best overall PSA response ((**D**), *p* < 0.005), presence of visceral metastases ((**E**), n.s.), presence of liver metastases ((**F**), *p* = 0.029), and PSMA-PET/CT response ((**G**), *p* < 0.001; SD stable disease, PD progressive disease, PR partial response). Additional values are given in Table 3.

Median OS was 15 months (95% CI 12.8–17.2 months). Median OS was stratified by clinical, haematological, and response criteria (VISION criteria fulfilled, three or more therapy cycles completed, presence of visceral metastases, presence of liver metastasis, haemoglobin levels ≥ 10 g/dL, AP, LDH, PSA best overall response, time to PSA progression, and PSMA-PET/CT response). Table 3 summarizes the median survival and significance of group differences stratified by these factors. Of those with three or more therapy cycles completed, haemoglobin levels ≥ 10 g/dL, AP, LDH, PSA best overall response, time to PSA progression, and PSMA-PET/CT response showed significant group differences. Exemplary Kaplan–Maier plots are shown in Figure 3 below.

### 3.6. Baseline Predictors for Improved Overall Survival

Baseline parameters can be used for optimizing patient selection for Lu-PSMA treatment, especially in cases where alternative therapies, such as taxane-based chemotherapy or PARP-inhibition, exist. Therefore, we analysed different clinical and laboratory variables that may interfere with prognosis in CRPC patients. Cox regression demonstrated that patients fulfilling the VISION criteria and patients with visceral or liver metastases had no significantly worse prognosis (Table 4). Patients with haemoglobin ≥ 10 g/dL lived significantly longer than patients with normal haemoglobin levels (HR 0.37, 95%CI 0.2–0.7, *p* < 0.01). Patients with higher baseline PSA value (≥116 ng/mL, mean in this cohort) had shorter overall survival (*p* = 0.03, HR 2.11).

## 4. Discussion

This study has been a single center retrospective data analysis of patients treated at a German Cancer Society certified Comprehensive Cancer Center. The study has shown that a personalized treatment approach with clearly defined criteria for patient selection using lower therapeutic Lu-PSMA dose as low as 2 GBq and not exceeding 6 GBq per therapy cycle and a variable treatment duration between 4 and 8 weeks can achieve comparable overall survival and response, as has been reported previously.

In the path-breaking VISION trial, mCRPC patients treated with Lu-177 PSMA 617 were found to have an overall survival of 15.3 months compared to 11.3 months for standard of care [6]. In another pivotal retrospective study, data from 270 patients treated in tertiary care hospitals in Australia, Germany, and the USA, among other retrospective studies, showed the median OS was around 13 months (range 9.2–16.5 months) [14]. The overall survival of 15 months (95% CI 12.8–17.2 months) in our study shows that an individualized approach to treatment is feasible without having any negative impact on survival, even in a real-world patient population, which typically has a high rate of study-ineligible patients.

Among several factors previously reported to have a negative impact on overall survival, visceral metastases have been reported consistently across the majority of the studies. However, in our patient population, the presence of visceral metastases was not found to have a significant impact on the overall survival, despite the high prevalence (46.3%). When analyzing the 18 patients with liver metastases separately, overall survival was statistically worse in the survival analysis when compared to patients without liver metastases (Figure 3), although during later follow-up, the Kaplan–Meier curves intercept again, and in Cox regression analysis for baseline parameters, the presence of liver or visceral metastases were also not independent predictors for reduced overall survival. We would therefore argue for patient selection based on PSMA expression profiles, and we did not offer Lu-PSMA therapy to patients with no PSMA expression on lesions visible on imaging. Previous studies have investigated the complementary effect of glucose metabolism imaging with 2-[18F]FDG-PET/CT in conjunction with PSMA-PET/CT as de-differentiated CRPC shift towards glucose metabolism, and patient selection was based on both imaging results. Although such preselected patients were found to respond better with Lu-PSMA therapy, their response did not convert into improved overall survival [7]. On the other hand, PSMA target heterogeneity [15,16,17] has been shown to be directly related to response and overall survival. Lower amounts of radiation dose in highly heterogeneous aggressive tumors can even be counterproductive. While patient selection based on FDG-PET in addition PSMA-PET might have a role, we would argue that PSMA heterogeneity based approaches are equally important, both intra- and inter-patient with diagnostic CT or MRI for correlation. As noted in the Materials and Methods section above, in case of discrepancy between PSMA-PET and CT, we sought to biopsy such lesions if amenable.

Clinical and laboratory parameters with dose adaptation seem to be at least equally important for patient selection, as the degree of serious adverse reaction was found to be less than previously reported in our study. Most notably, grade 3 or a higher grade of anemia was found to occur in only 14/86 (16.3%) compared to 12.9% observed in the VISION trial and similar to a recently published study with 7.3% [18]. Thrombocytopenia grade 3 or higher was noted in 4.7% of the patients in our study compared to 7.9% in the VISION trial. This difference could be related to the difference in both the patient populations and the design of the study. Unlike the VISION trial, where cut-off for hemoglobin and platelets were 9 g/dL and 100 × 10^9^/L, respectively, we treated patients with hemoglobin and platelets up to 6 g/dL and 50 × 10^9^/L. Despite treating patients with significantly decreased baseline bone marrow reserve, the overall survival was still comparable. However, this positive trend in favor of the comparable severity of higher-grade toxicity in individualized treatment approaches with significantly lower bone marrow reserve was also reflected in the frequency of fatigue as well as dry mouth of any grade. 

The individualized approach for Lu-PSMA therapy has limitations, though, as it requires intensive patient monitoring and careful patient selection in tumor boards. More-over, lower therapeutic doses of Lu-PSMA, as we used in our study, could potentially negatively impact the response rate without effective disease control. However, 50% PSA reduction was observed in approximately 40% of patients, which is similar to the 43–45% that was reported in the report of Gafita et al. [14]. More importantly, patients who showed at least 50% PSA reduction had significantly better overall survival, and this is line with several other previously reported studies.

Anemia has been found to be one of the negative predictors of overall survival [14,19], and we also noted that anemia has a negative impact on survival in our study. There are several possible reasons for this association. Patients with advanced bone marrow disease tend to have anemia, but they are also likely to have lower overall survival from the outset. Anemic patients are generally treated with a reduced dose or number of cycles of Lu-PSMA, which can, theoretically, reduce the survival probability. This theory was further supported in our study, and was also supported in the data from 270 patients in Gafita et al. [14], where patients completing three therapy cycles and receiving approximately 18 GBq of treatment were found to survive longer (Figure 3). 

The tumors of patients treated with Lu-PSMA undergo therapeutic pressure, as is true with other cytotoxic treatments as well. This is why it is essential to identify prognostic markers in order to intervene and to select patients at an early stage who are unlikely to profit from continuing Lu-PSMA therapy. In our study, we observed that patients who are not achieving a 50% reduction in PSA at 60 days post first therapy cycle tended to have shorter overall survival (Figure 3). This potential negative predictive marker should be further investigated in prospective trials. While the ideal time point to assess PSA response after Lu-PSMA therapy and the ideal approach in prostate cancer therapy in general have both been a subject of debate for quite some time [20], it is generally accepted that an early increase in PSA values for at least 4 weeks can reflect tumor cell degradation, which reflects, among other factors, the flare phenomenon observable on bone scintigraphy. Thus, we assessed PSA values after 4 weeks, 8 weeks, and sometimes, additionally, 12 weeks after therapy, and we chose the PSA value after 8 weeks to define PSA response.

Due to its retrospective nature, our study has several limitations. Patient selection was made on an individualized basis in the tumor board and the therapeutic Lu-PSMA dose was defined based on total tumor mass and laboratory values, and, therefore, the heterogeneity within the group was substantial. However, the results demonstrate that this individualized approach does not result in inferior patient survival compared with prospective trials and other retrospective analyses. Another limitation of our study is that we did not look at the impact of the patient selection criteria on the overall survival of patients that were excluded from getting Lu-PSMA therapy, and, thus, we did not have a control arm. However, the therapeutic effectiveness of Lu-PSMA compared to other therapeutic regimens was beyond the scope of this study.

## 5. Conclusions

This study emphasizes that a personalized approach to radioligand therapy with [177Lu]-Lutetium-PSMA is safe, feasible, and effective. Our retrospective analysis identified markers that can potentially be used for further patient stratification, and it showed that the presence of visceral metastasis is not associated per se with poor overall survival, while higher haemoglobin levels and lower PSA values at the start of therapy seem to be favourable concerning overall survival. These factors should be considered for future prospective treatment trials.

## Figures and Tables

**Table 1:** Patient characteristics (median with range or number and percent).

Age (*n* = 86)—Years	71 (52–95)
ECOG (*n* = 86)	
• ECOG 0	36 (41.9%)
• ECOG 1	34 (39.5%)
• ECOG 2	14 (16.3%)
• ECOG 3	2 (2.3%)
PSA at Treatment Start (*n* = 83)—ng/mL	116 (0.19–3160)
GFR (*n* = 86)—mL/min	85 (14–123)
• GFR < 60 mL/min	13 (15.1%)
• GFR ≥ 60 mL/min	73 (84.9%)
Alkaline Phosphatase (*n* = 84)—U/L	117 (31–1764)
Lactate Dehydrogenase (*n* = 82)—U/L	244 (103–2500)
Gleason-Score (*n* = 86):	
• Score ≤ 6	4 (4.7%)
• Score 7	21 (24.4%)
• Score 8	22 (25.6%)
• Score 9	30 (34.9%)
• Score 10	2 (2.3%)
• Unknown	7 (8.1%)
Previous local therapy (*n* = 86)	
• Prostatectomy	46 (53.5%)
• Radiation	6 (7.0%)
• TURP	8 (9.3%)
• No local therapy	26 (30.2%)
Previous new hormonal agent (*n* = 86)	Yes: 81 (94.2%) No: 5 (5.8%)
Previous taxane therapy (*n* = 86)	Yes: 65 (75.6%) No: 21 ^1^ (24.4%)
Site of disease (baseline PET/CT) (*n* = 86)	
• Lymph Node Metastases	Yes: 60 (70.0%) No: 26 (30.0%)
• Bone Metastases	Yes: 79 (91.9%) No: 7 (8.1%)
• Visceral Metastases	Yes: 39 (45.3%) No: 47 (54.7%)
of those:	
Liver metastases	18 (46.2%)
Adrenal gland	14 (35.9%)
Lung	10 (25.6%)
Peritoneal	4 (10.3%)
Brain	1 (2.6%)

^1^ No previous taxane therapy due to hematologic limitations (*n* = 13) or patient refused chemotherapy (*n* = 8).

**Table 2:** Summary of Adverse Events under Lu-PSMA therapy.

Adverse Event	All Grades	Grade ≥ 3
Any adverse event	71	23
Anemia	71	14
Leucopenia	51	13
Thrombocytopenia	30	4
Nausea	15	
Dry mouth	13	2
Difficulty voiding urine	11	3
Fatigue	9	1
Vomiting	8	
Bone pain	7	
Decreased appetite	1	
Constipation	1	
Diarrhea	1	
Peripheral edema	1	
Headache	1	

**Table 3:** Survival Analysis—Kaplan–Maier.

Factor	Groups	*n*	Median Survival in Months	95% Confidence Interval in Months	Chi-Square Log Rank (Mantle-Cox)	*p*-Value
VISION criteria met	Yes	60	13	8–18	2.1	0.151
	No	24	16	12–20		
≥3 therapy cycles	Yes	50	23	19–27	33.3	<0.001 *
	No	36	5	3–7		
Visceral metastases	Yes	38	15	11–19	2.0	0.152
	No	44	15	12–18		
Liver metastases	Yes	18	6	4–8	4.8	0.029 *
	No	68	16	13–17		
Haemoglobin	≥10 g/dL	58	19	12–26	19.5	<0.001 *
	<10 g/dL	28	5	1–9		
AP	Normal	44	18	13–23	7.9	0.005 *
	Elevated	40	9	4–14		
LDH	Normal	42	18	13–23	5.6	0.018 *
	Elevated	40	9	3–15		
PSA at start of therapy above median	≥116 ng/mL	43	9	3–15	10.5	0.001 *
	<116 ng/mL	40	23	14–32		
PSA best overall response	≤50%	44	12	7–17	7.7	0.005 *
	>50%	29	23	18–28		
Time to PSA progression	≤60 days	22	8	5–11	29.6	<0.001 *
	>60 days	51	23	19–27		
PSMA-PET/CT response	PR	27	23	19–27	16.8	<0.001 *
	SD	11	15	0–32		
	PD	24	10	7–13		

* *p*-Values with significant group differences.

**Table 4:** Multivariate Cox regression analysis for baseline parameters.

	*p*-Value	HR	95% CI
VISION Criteria met	0.98	1.01	0.5–2.0
Presence of Visceral metastasis	0.46	1.31	0.6–2.7
Presence of Liver metastasis	0.26	1.58	0.7–3.5
Pre-treatment Haemoglobin ≥ 10 g/dL	<0.01 *	0.37	0.2–0.7
Pre-treatment PSA ≥ 116 ng/mL	0.03 *	2.11	1.1–4.1

* *p*-value considered significant, HR = hazard ratio, CI = confidence interval.

## Data Availability

The data that support the findings of this study are available from the corresponding author upon reasonable request.
